# MOTS-c, the Most Recent Mitochondrial Derived Peptide in Human Aging and Age-Related Diseases

**DOI:** 10.3390/ijms231911991

**Published:** 2022-10-09

**Authors:** Zahra Mohtashami, Mithalesh K. Singh, Nasim Salimiaghdam, Mustafa Ozgul, M. Cristina Kenney

**Affiliations:** 1 Department of Ophthalmology, Gavin Herbert Eye Institute, University of California Irvine, Irvine, CA 92697, USA; 2 Department of Pathology and Laboratory Medicine, University of California Irvine, Irvine, CA 92697, USA

**Keywords:** MOTS-c, mitochondrial derived peptides, mitochondrial dysfunction, aging, age-related diseases

## Abstract

MOTS-c, a 16 amino acid mitochondrial derived peptide, is encoded from the 12S rRNA region of the mitochondrial genome. Under stress conditions, MOTS-c translocates to the nucleus where it regulates a wide range of genes in response to metabolic dysfunction. It is colocalized to mitochondria in various tissues and is found in plasma, but the levels decline with age. Since MOTS-c has important cellular functions as well as a possible hormonal role, it has been shown to have beneficial effects on age-related diseases including Diabetes, Cardiovascular diseases, Osteoporosis, postmenopausal obesity and Alzheimer. Aging is characterized by gradual loss of (mitochondrial) metabolic balance, decreased muscle homeostasis and eventual diminished physical capability, which potentially can be reversed with MOTS-c treatment. This review examines the latest findings on biological effects of MOTS-c as a nuclear regulatory peptide and focuses on the role of MOTS-c in aging and age-related disorders, including mechanisms of action and therapeutic potential.

## 1. Introduction

Metabolism is a crucial biological function that consists of catabolic and anabolic reactions in living cells [1]. Multiple interrelated metabolic processes such as glycolysis, citric acid cycle, oxidative phosphorylation, fatty acid-oxidation, and gluconeogenesis provide energy for cells to grow, reproduce, and preserve their structures [2,3].

The mitochondria, complex organelles with endosymbiotic origins in early eukaryotic cells, convert most energy via oxidative phosphorylation (OXPHOS), the citric acid cycle, and fatty acid oxidation [1]. Mitochondria are involved in amino acid, lipid, nucleotide, apoptotic, calcium, and retrograde signaling [4,5]. The fact that mitochondria have their own genome, mitochondrial DNA (mtDNA), supports this notion [4].

Mitochondria are dynamic organelles that are responsible for metabolism and the conversion of energy-storing molecules, such as ATP, for the function of the cell [4]. However, mitochondria communicate via reactive oxygen species (ROS), Ca2+, and cytochrome C [5,6,7]. Given their importance, it is not unexpected that mitochondria are sensitive to intrinsic stressors, including mutation and deletion of mtDNA [8], a lack or excess of energetic substrates [9], an increase in ROS levels [10], and stressor extrinsic agents such as toxins, viruses, bacteria, and ultraviolet rays [11]. Chemicals can change mitochondrial function and dynamics, causing aging, neurological illness, diabetes, and cancer [8]. Furthermore, it is believed that mitochondria are substantially capable of locally generating systemic reactions [4]. According to new research, mitochondria have an enlarged genetic impact with the discovery of the Mitochondrial Derived Peptides (MDPs). Humanin (HN), small HN-like peptides (SHLPs), and mitochondrial open Reading frame (ORF) of the twelve S-c (MOTS-c) are MDPs that can modulate cellular metabolism and provide cytoprotection, shattering paradigms with respect to the previously recognized mitochondrial activity [4,5].

**Figure 1:**
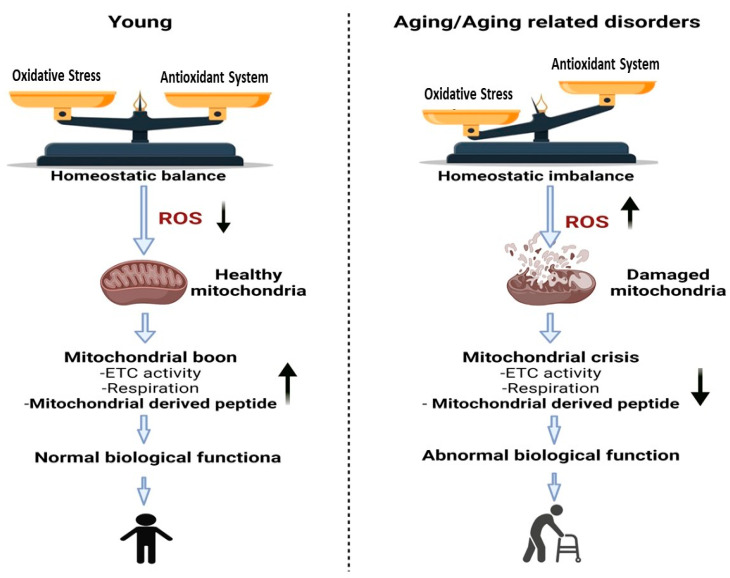
Changes that occur in mitochondria due to aging or aging related disorders are associated with a reduction in the function of mitochondria. Due to the accumulation of mutations and the oxidative damage generated by reactive oxygen species (ROS), the mitochondrial DNA volume, integrity, and functionality all decline with advanced. Left Panel represents young individuals with balanced homeostasis leading to normal biological functions of tissues. Right Panel represents greater levels of oxidative stress that lead to increased ROS and mitochondrial dysfunction along with abnormal biological functions.

Few studies have examined the mitochondrial responses under controlled stress, such as physical stress. There are considerable data demonstrating that stress events are involved in the regulation of this novel class of peptides [4,5]. Aging is characterized by gradual loss of (mitochondrial) metabolic balance, elevated ROS levels and eventually diminished physical capability (Figure 1) [6,7]. Indeed, aging is a substantial risk factor for a variety of chronic non-infectious diseases [8,9,10]. This review examines the biological effects of MOTS-c as a nuclear regulatory peptide and focuses on the role of MOTS-c in aging and age-related disorders, including mechanism of action and therapeutic potential.

## 2. What Are Mitochondrial Derived Peptides (MDPs)?

Mitochondrial-derived peptides (MDPs) are translated peptides encoded by short open reading frames (sORFs) within known mitochondrial (mt) DNA genes. The MDPs have cytoprotective roles in preserving mitochondrial function and cell viability under stress conditions [4,5,6,7]. The mammalian mtDNA encodes 13 mRNAs, 22 tRNAs, and 2 rRNAs (12S & 16S rRNA) which are structural components of the electron transport chain [8,9]. To date, eight MDPs have been identified, all of which are transcribed from sORFs found in mtDNA genes that encode from the 12S rRNA and 16S rRNA transcripts [4].

The 16S ribosomal RNA gene is 1559 nucleotides in length, found within the MT-RNR2 gene and spans mtDNA nucleotide pairs (nps) 1671–3229 [10]. The 16S rRNA region encodes for Humanin, the first well-studied MDP and Small Humanin-Like Peptides (SHLPs) [11].

**Figure 2:**
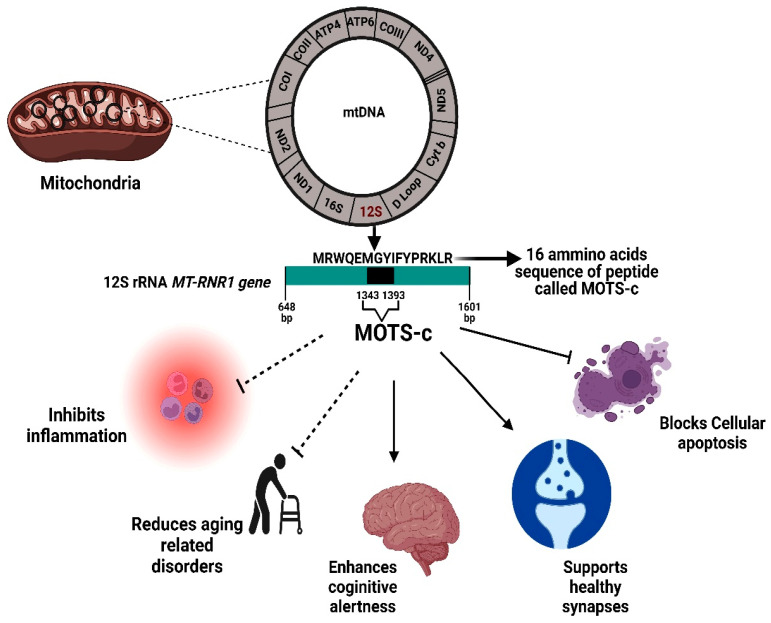
Physiological significance of the MOTS-c protein. MOTS-c is encoded from a region within the 12S rRNA MT-RNR1 gene. The MOTS-c protein has both inhibitory effects (inflammation, age-related disorder, apoptosis) and also promotes healthy functioning in brain and other tissues. bp, base pair.

The 12S rRNA gene (*MT-RNR1* gene) is 954 nps, spanning from 648 to 1601 nps, which represents approximately 6% of total mtDNA. This 12S rRNA region encodes for MOTS-c (mitochondrial open reading frame of the 12S rRNA type-c), the most recently identified MDP. The discovery of HN, SHLPs and MOTS-c peptides has led to novel areas of research because of their origin from the mitochondrial genome, and subsequent revelations that these peptides play critical functions of neuroprotection, metabolism, signaling and inhibition of apoptosis. Beside some common overlapping functions, each MDP has its own exclusive role causing different response [5]. MOTS-c role in various pathophysiological conditions is described in (Figure 2).

### 2.1. MOTS-c: Origin, History, and Structure

After the discovery of humanin (HN) in 2001, researchers went on in 2015 to identify another new mitochondrial derived peptide (MDP) known as MOTS-c [4]. MOTS-c is in a variety of tissues, co-localizes to mitochondria, and is found in plasma of rodents and humans. MOTS-c has important cellular functions as well as a possible hormonal role [4,12].

In order to replicate complementary DNAs (cDNAs) used to map the region containing 12S rRNA, human myeloblasts were stimulated by interferon [13]. Careful analyses of the sORFs within the human 12S rRNA revealed one consisting of 51 base pairs which is translated into a 16 amino acid sequence of peptide (MRWQEMGYIFYPRKLR) termed as MOTS-c [14]. It was ultimately demonstrated that the MOTS-c peptide was not of nuclear DNA origin (possibly a nuclear mitochondria DNA transfer, NUMT), but rather completely homologous to the mtDNA genome [4].

The mitochondrial genome evolves at a faster rate than the nuclear genome, owing to a greater mutation rate and clonal propagation, which can result in sequence alterations between closely related species [10,15,16]. However, due to a significant positive selection force, some regions of 12S and 16S rRNA are largely maintained across species [17]. MOTS-initial c’s 11 amino acid residues (for a total of 16 amino acids) are highly conserved across 14 mammalian species [4,17]. Notably, “dwarf” sORFs that encode for peptides of 20 amino acids are less conserved [18], which could explain why MOTS-c is not conserved in some lower eukaryotes such as C. elegans and Drosophila melanogaster [19,20].

### 2.2. Molecular Mechanisms and Pathways of MOTS-C

Mitochondrially derived peptides (MDPs) are retrograde signaling molecules. These peptides regulate mitochondrial bioenergetics and metabolism, which in turn alter systemic insulin sensitivity and glucose homeostasis. Furthermore, Kim et al. demonstrated that MOTS-c, a mitochondrial-encoded peptide, may dynamically translocate to the nucleus in response to metabolic stress and modulate adaptive nuclear gene expression [21]. Humanin and MOTS-c are the two most commonly studied MDPs. Humanin receptors include the seven transmembrane G-protein-coupled receptor formyl-peptide receptor-like-1 (FPRL1) and a trimeric receptor that includes the ciliary neurotrophic factor receptor (CNTFR), the cytokine receptor WSX-1, and the transmembrane glycoprotein gp130 (CNTFR/WSX-1/gp130) [22]. While to date there have not been any cellular receptors described for the MOTS-c peptide. MOTS-c release in the blood is also termed as “mitochondria hormone” or “mitokine” [23]. Its circulation is regulated by the folate cycle and signaling via cAMP and AMPK [4]. MOTS-c expression is age-dependent [24].

MOTS-c is an important regulator for energy balance and is highly associated with amino acid, carbohydrates, and lipid metabolism. In mammalian cells, it is encoded from the mitochondrial DNA and under stress conditions, it then translocates to the nucleus, which is accompanied by higher ROS production [21]. The MOTS-c nuclear translocation is 5′-adenosine monophosphate-activated protein kinase (AMPK) dependent [23,25]. MOTS-c triggers the activation of AMPK and accumulation of 5-aminomidazole-4-carboxamide ribonucleotide (AICAR), a known AMPK activator, by inhibiting the folate cycle and de novo purine biosynthesis [7,26].

AMPK is the major sensor and key regulator of cellular metabolism based on energy availability [27]. Upon rise in the ATP:ADP or ATP:AMP ratios, AMPK is activated and alters the metabolism toward catabolism induction and anabolism suppression by phosphorylation of crucial proteins in various pathways, including mTOR complex 1 (mTORC1) [28,29].

Additionally, during stress, AMPK activates Peroxisome proliferator-activated receptor Gamma Co-activator-1α (*PGC-1α*) via direct phosphorylation [30,31]. The *PGC-1α* regulates expression of antioxidants in mitochondria and is a key factor in mito-nuclear communication. It may interact with Nuclear Factor, Erythroid -1 and -2 (*NRF-1/2*) to block mitochondrial oxidative stress, promote the clearance of damaged mitochondria and enhance mitochondrial biogenesis [32].

**Figure 3:**
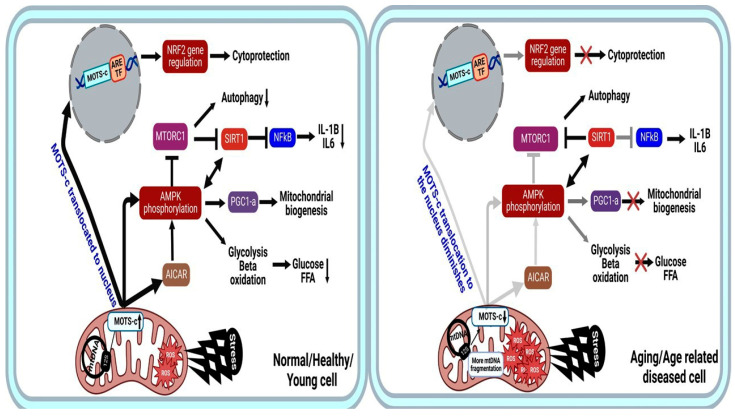
MOTS-c mechanism of action in normal young healthy state (**left**) vs. Aging or age-related diseases (**right**). The peptide is capable of interacting with the nuclear genome to provide cryoprotection and has beneficial effects mainly when it comes to the regulation of the metabolisms of AMPK and AICAR. Faded lines indicate less dependent effect or production, Faded line with cross indicate signal is completely lost, and cross indicates signal is lost. AMPK, 5′-Adenosine Monophosphate-activated Protein Kinase; AICAR, 5-AminoImidazole-4-CarboxAmide Ribonucleotide; FFA, Free Fatty Acid; FFA-B, Free Fatty Acid-B oxidation; ARE, Antioxidant Response Elements, TF-Transcription factor.

In the nucleus, MOTS-c regulates a wide range of genes in response to metabolic dysfunction, including those containing antioxidant response elements (ARE) [33]. It interacts with ARE-regulating stress-responsive transcription factors, such as Nuclear Factor Erythroid 2-Related Factor 2 (*NFE2L2/NRF2*) [8,25,34]. *NFE2L2/NRF2* is a stress-responsive transcription factor that responds to ROS and protect cells under oxidative stress [35]. NRF2/ARE pathway activation plays an antioxidative role in treating acute kidney injury and vascular dysfunction. Notably, *NRF2* intersects with AMPK [35] and can regulate MOTS-c-related metabolic pathways. The MOTS-c/NRF2 relationship boosts mitochondrial protection genes, and MOTS-c overexpression increases NRF2 signaling [32]. Different metabolic pathways of MOTS-c are summarized in (Figure 3).

During rest periods, the MOTS-c peptide has a mitochondrial-association and only low quantities of endogenous MOTS-c are found in the nucleus [25]. The distinct MOTS-c nuclear regulatory feature sets it apart from all other MDPs and makes it a promising agent for future research in the fields of diagnosis and treatment of a broad range of metabolic diseases, including aging-related disorders.

### 2.3. Aging and Longevity: MOTS-c

Aging is a lifelong process that leads to senescence, or a breakdown of biological functions and an incapacity to respond to metabolic stress [33]. Improved mitochondrial fitness and physical capacity aid healthy aging.

MOTS-c levels in 70–81-year-olds drop by nearly 21% compared to 18–30-year-old individuals [36]. MOTS-c shares metabolic pathways with age-modifiers. NAD+, a metabolic cofactor in redox reactions and a critical modulator of cell signalling and survival pathways, diminishes with age. Moreover, NAD+ as a potent sirtuin activator, plays a key role in energy metabolism, cell survival, and aging in model species, therefore, maintaining its level could postpone age-related disorders and, in certain cases, increase longevity [13,37,38,39].

MOTS-c (i) elevates NAD+ levels, (ii) has glycolytic effects via sirtuin 1 (*SIRT1*) [7], (iii) influences the folate/methionine cycle and (iv) restricts methionine metabolism. Methionine shortage extends mouse lifespan by 45%, lowers visceral fat and age-related diseases, and prevents lens degeneration [40,41].

MOTS-c, whose levels decline with age, has a wide range of health-span consequences. In vivo mice studies showed that intraperitoneal (IP) MOTS-c (15 mg/kg/day) improved the physical performance of mice of different ages (2, 12, 22 and 23.5 months) over a two-week period. This treatment improved the physical capacity and slowed the emergence of age-related deficits [24,42].

Loss of dermal collagen is responsible for aging skin’s flattened dermo-epidermal interface and disorganized extracellular matrix [43,44]. Li et al., reported that MOTS-c (synthesized, 10 mg/kg, intraperitoneal, 6-week-old mice) increased skin collagen by reducing IL-6, a key inflammatory factor in matrix metalloproteinase 1 (*MMP1*) and collagen loss in the dermis. MOTS-c may prevent skin aging by lowering inflammation, which leads to an increase of dermal collagen [32].

**Figure 4:**
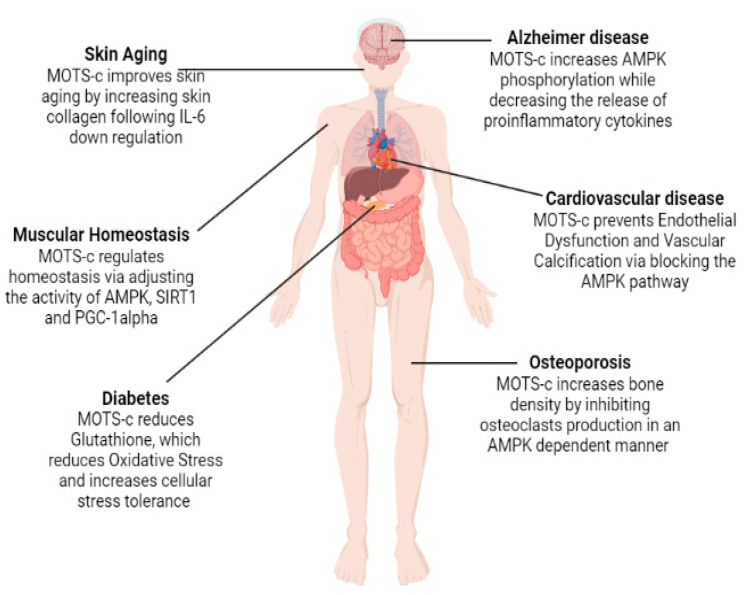
Role of MOTS-c in relation to different age-related disorders.

While the process of aging is linked to several different factors, including shifts in metabolic control, altered gene expression patterns [45], and high production of ROS [46,47,48], it is unclear exactly how these factors interact to cause aging. Therefore, older age is the biggest risk factor for chronic diseases and functional impairments that limits life expectancy [49]. MOTS-c indications in aging and several age-related diseases are presented schematically in (Figure 4).

### 2.4. Age-Related Diseases: MOTS-c

#### 2.4.1. Diabetes

Diabetes is spreading at the rapid rate around the world, and its complications are a leading cause of death [50,51]. Oxidative stress is a critical factor in the development, progression, and consequences of diabetes [51]. MOTS-c increases glucose clearance, lactate levels in culture media, and intracellular glucose levels [4,24,30].

Type 1 diabetes (T1D) is an autoimmune condition that destroys insulin-secreting b cells [52,53]. Increasing autoreactive T cell glycolysis is crucial in preventing and treating autoimmune diseases [54]. Mitochondria, the main metabolic organelle, regulate T cell activation and differentiation [55,56]. Pugliese et al., demonstrated that systemic MOTS-c therapy in NOD (non-obese diabetic) mice postpones diabetes and improves blood glucose levels. It lowers islet infiltration and insulitis in NOD mice [57]. In another study, 17-week-old male mice fed a high-fat diet were injected intraperitoneally with 2.5 mg/kg of synthetic form of MOTS-c (Genscript) twice a day for three days. MOTS-c–injected mice showed lower glucose and insulin levels than controls, suggesting it enhances insulin sensitivity in high-fat diet-induced obese mice. Plasma oxidized glutathione was lower in the MOTS-c injected group, which may be linked to reduced oxidative stress in cells and enhanced cellular stress resistance [58].

Type 2 diabetes (T2D) causes insulin insufficiency due to targeted tissue resistance [59]. Mitochondrial dysfunction is linked to T2D and its complications [60]. In a study of 225 normal and pre-diabetic patients, MOTS-c levels were considerably lower in T2D than controls subjects. MOTS-c has a negative correlation with age, HbA1c, and glucose [58,61]. Asian-specific mtDNA polymorphism m.1382A>C leads to a K14Q amino acid replacement in MOTS-c and is associated with increased T2D susceptibility in men. Controversial studies linked the m.1382A>C polymorphism to the Japanese people’s remarkable lifespan, which was eventually corrected in a larger cohort of study participants [62].

Gestational diabetes mellitus (GDM) is glucose intolerance, insulin resistance and deficiency early in pregnancy [63,64]. It affects 10.5–24.2% of pregnancies globally [65]. It can lead to obesity, type 2 diabetes, and other metabolic disorders [66]. A GDM mouse model (8 weeks old) was generated to study the effects of MOTS-c during pregnancy. The MOTS-c intraperitoneal injections (10 mg/kg) targeted the skeletal muscle and lead to reduced birth weight and GDM-related infant mortality [67].

#### 2.4.2. Cardiovascular Diseases

Cardiovascular disease (CVD) refers to functional and structural aberrations in small coronary vessels [68,69] and is one of the most prevalent diseases that causes high morbidity and mortality around the world particularly in aged populations [70,71].

Endothelial Dysfunction (ED), a type of CVD, shifts the endothelium toward inflammation and reduces vasodilation [72,73]. Qin et al. found a correlation between reduced MOTS-c levels and coronary endothelial dysfunction (ED) in humans. Pre-treating rat or RAS mice aortic explants with MOTS-c increased acetyl choline-mediated relaxation [74]. It also suppressed the expression of pro-inflammatory cytokines (TNF-α, IL-6, IL-1β) [75] by inhibiting the AMPKs signalling pathway [76,77]. These data suggest that MOTS-c may prevents ED via inhibiting the AMPK/NF-κB pathway [74].

Vascular calcification (VC) is the abnormal deposition of calcium phosphate crystal deposits in artery walls. It complicates the progression of chronic kidney disease, cardiac valve disease, and atherosclerosis [78]. Wei et al. demonstrated that MOTS-c therapy prevented vascular calcification in Vitamin D3 plus nicotine-treated rats (VDN) by activating AMPK signalling, and reversing the overexpression of Angiotensin 1(AT-1) and Endothelin B (ET-B) receptors induced by VDN [78,79]. Overexpression of AT-1 and ET-B receptors is linked to increased myocardial fibrosis and cardiac dysfunction [80], which is consistent with the beneficial effect of MOTS-c against both oxidative stress and development of myocardial contractile dysfunction [81]. The findings show that MOTS-c may serve as a VC inhibitor by activating the AMPK signalling pathway and inhibiting the AT-1 and ET-B receptors [79,82,83].

Identifying non-invasive blood-based biomarkers is crucial for early diagnosis and prognosis of many diseases. Studies have supported evaluation of novel blood indicators for cardiometabolic dysfunction [84]. Thus, MOTS-c could be an early predictor of coronary atherosclerosis and a possible therapeutic marker in ED.

#### 2.4.3. Post-Menopausal Disorders

Postmenopausal women have lower ovarian hormone production and a higher risk of metabolic dysfunction, including reduced energy expenditure, obesity, and impaired insulin secretion and sensitivity [85,86]. In obesity, fat excess impairs adipose function and increases fatty acids and systemic inflammation [87]. In a post-menopause model of ovariectomized mice, the administration of MOTS-c lead to increased brown fat activation and inflammatory markers in white adipose tissue, lower fatty acid levels in serum and liver, along with limited weight gain [88]. MOTS-c activated AMPK to minimize fat deposition, restore energy, and improve insulin sensitivity [89,90].

#### 2.4.4. Osteoporosis

Low bone mass and altered microarchitecture lead to bone fractures in osteoporosis patients [91]. The imbalance between synthesis and absorption of bone inorganic minerals and organic matrices, especially Type I collagen, is crucial in Osteoporosis [91,92]. Type I collagen (*COL1A2*) makes up 80–90% of bone organic matter [92]. In type I collagen synthesis and metabolism, *TGF-β* (transforming growth factor-β) stimulates cell proliferation, differentiation, and immigration [92,93]. The hFOB1.19 (Human Fatal Osteoblastic) cells treated with MOTS-c showed higher expression levels of *TGF-β*, *SMAD7*, and *COL1A2* at both mRNA and protein levels. These findings demonstrated that MOTS-c induced osteoblasts to produce type I collagen via the *TGF-β/SMAD* pathway [94]. BMSCs (Bone mesenchymal stem cells) differentiation, which is also dependent on *TGF-β* and *SMAD*, is another attractive target. BMSCs treated with MOTS-c had increased expression levels of *TGF-β1*, *TGF-β2* mRNA and proteins [95].

Postmenopausal osteoporosis causes bone resorption due to oestrogen deprivation [96,97]. Osteoclasts are the only cells that can resorb bone, and RANKL is their essential cytokine [77,98,99]. AMPK regulates RANKL-induced osteoclast differentiation [100,101]. It increases osteoblast proliferation, differentiation, and mineralization and reduces apoptosis [102]. MOTS-c treatment significantly reduced bone loss in 8-week-old C57BL/6 females mice with ovariectomies by suppressing osteoclast formation in an AMPK-dependent way [103].

The model of Ultra-High Molecular Weight Polyethylene Particles (UHMWPE) has wear-induced osteolysis, bone loss that causes loosening of implants and peri-implant fractures [104,105]. This bone loss is brought on by excessive bone resorption and inadequate bone synthesis as well as elevated expression levels of *TNF-α*, *IL-1*, and *IL-6* caused by UHMWPE particles. Immunofluorescence assays show that MOTS-c treatment downregulated the macrophages which were contributing in inflammatory reactions. Furthermore, MOTS-c injection into the area where UHMWPE particles were implanted, prevented significant bone mass loss and substantially reversed bone loss [77,106]. MOTS-c is a promising osteoporosis treatment since it enhances bone density, volume ratio, and cell quantity via a variety of mechanisms.

#### 2.4.5. Alzheimer’s Disease

Aging is a major risk factor associated with Alzheimer’s disease (AD). Damaged mitochondria produce reactive free radicals, which cause oxidative stress, cell death and cognitive impairments in AD [107,108,109] Chang et al., showed MOTS-c therapy increased the formation of object and location recognition memories by phosphorylating AMPK, decreasing astrocyte and microglia activation and lowering proinflammatory cytokine production [110].

### 2.5. MOTS-c in Relation to Muscle Homeostasis and Physical Activity

Mitochondria nourish skeletal muscle during exercise and communicate exercise-induced signals to other organs [4,39,73,74,75,76]. MOTS-c and exercise training had an additive effect on boosting PGC-1α gene expression [30]. MOTS-c treatment in mice activated skeletal muscle AMPK, a well-known exercise regulator, by elevating cellular levels of AICAR (an AMPK agonist) and *GLUT4* [31,44]. Regular aerobic activity, such as treadmill workouts, increased *PGC-1α*, *GLUT4* expression, and AMPK phosphorylation levels in wild-type mice [30]. MOTS-c modulates exercise-sensitive signalling intermediates (AMPK, SIRT1, and PGC-1α) in skeletal muscles during exercise [23,24], resulting to fatty acid oxidation and mitochondrial biogenesis [4]. Exercise reduces the incidence of T2D and CVD, which also increase mortality risk [111,112].

Li et al., MOTS-c therapy and regular aerobic exercise for 8 weeks altered 98 and 47 pathogenic genes, respectively, with 24 genes related to angiogenesis, inflammation, and apoptosis overlapping. These data imply that both MOTS-c and exercise reduce diabetic heart dysfunction through similar pathways [113].

## 3. Future Implications

The accumulation of mtDNA mutations, as well as the resultant metabolic dysfunction, are both deeply involved in the aging process [114,115]. We hypothesize that these mitochondrial genetic changes may contribute to the age-dependent decrease in MOTS-c levels [10]. This would add another dimension to the importance of mitochondrial homeostasis during aging and the emerging, exciting biology of MDPs.

Therefore, a malfunction/degradation of mtDNA, which is associated with aging, may cause not only a direct decrease in mitochondrial function but also a progressive loss of expression of MDPs, which would reduce the regulatory peptide activities of MDPs. This may occur because of the progressive degradation of mtDNA. Given that aging is associated with a decline of mitochondrial functions concurrently with the development of aging-related diseases, such as diabetes and metabolic syndrome, and given that the tissue and circulating levels of MOTS-c fall with age, it is compelling to hypothesize that declining MDP levels are also related to age-related metabolic deterioration.

Age-modifiers and MOTS-c share metabolic pathways. NAD+ is a metabolic coenzyme involved in redox activities that declines with age. Increasing its levels can improve age-related disorders [1,2]. NAD+ is also a significant activator of sirtuins, which regulate aging and age-related disorders in yeast to mammals [37,116]. MOTS-c elevates intracellular NAD+, and *SIRT1* mediates its glycolytic actions [23]. MOTS-c inhibits the folate/methionine cycle, reducing methionine metabolism. In rodents, methionine restriction can extend lifespan by 45%, limit age-related illnesses (e.g., cancer), postpone lens degeneration, reduce visceral fat, and boost GSH [38,39,117].

We hope that by bringing attention to the potential therapeutic value of MOTS-c in the treatment and diagnosis of the age-related life-threatening morbidities, this review will encourage more clinical research into the response of this new class of peptides to age-related treatments. This review will also raise awareness of the potential therapeutic values of MOTS-c.

## Data Availability

Not applicable.
